# Applications of multi-walled carbon nanotube in electronic packaging

**DOI:** 10.1186/1556-276X-7-183

**Published:** 2012-03-09

**Authors:** Cher Ming Tan, Charles Baudot, Yongdian Han, Hongyang Jing

**Affiliations:** 1 School of Electrical and Electronic Engineering, Nanyang Technological University, Jurong, 639798, Singapore; 2 ST Microelectronics Asia Pacific Pte Ltd, 7 Serangoon North Avenue 5, Serangoon, 554812 Singapore; 3 School of Materials Science and Engineering, Tianjin University, Tianjin, 300072, China

## Abstract

Thermal management of integrated circuit chip is an increasing important challenge faced today. Heat dissipation of the chip is generally achieved through the die attach material and solders. With the temperature gradients in these materials, high thermo-mechanical stress will be developed in them, and thus they must also be mechanically strong so as to provide a good mechanical support to the chip. The use of multi-walled carbon nanotube to enhance the thermal conductivity, and the mechanical strength of die attach epoxy and Pb-free solder is demonstrated in this work.

## Introduction

Electronic packaging is to protect and cool the microelectronic chips as well as to provide electrical and mechanical connections between the chip and the outside world. It controls the chips' electrical performance, size, cost, and reliability. As the power densities of microelectronic chips are increasing with faster and denser circuits on the chips, heat dissipation of the packaging becomes critical in determining their reliability and performances as both the high temperature and the associated large thermo-mechanical stress within the packages can degrade the circuit performances and the lifetime of the chips. As most of the heat generated by the chips is dissipated via the die attach material and solders, the thermal and mechanical properties of the die attach material and solder should be improved with technology, and the use of fillers is a common strategy employed. For example, a key strategy to enhance the electrical and thermal properties of dielectric polymers is to add metal particles into it [1,2].

Among the different types of filler used in composite materials, the only one having exceptionally high thermal conductivity [3-17] and unique mechanical properties [18,19] is the carbon nanotube [CNT]. However, exploiting CNT is not always an obvious task. What may appear as an advantage at one time may turn out to be a major disadvantage in another circumstance. For instance, the chemical inertness of CNT is a very interesting property which ensures that the material does not change or degrade with time. Conversely, this same property becomes quite troublesome when the solubility and anchoring of the fillers in the host matrix are concerned.

In this paper, we report our experimental work on the development of chemical treatments by grafting the molecules on the surface of multi-walled CNT [MWNT], and uniform dispersion with good interface transfer is obtained. Using this treated CNT, we show an improvement in the thermal and mechanical properties of epoxy, and this can be applied to die attach material to enhance heat dissipation from the chip to the lead frame and/or substrate. We also report another experiment where we embedded Ni-coated CNT into lead-free solder to enhance its properties.

### Chemical treatment of MWNT for epoxy applications

Traditionally, to debundle CNTs, they are put in an aprotic, polar solvent such as dimethylformamide or *N*-methylpyrrolidone and sonicated for some hours [20-23]. However, the limit of such a strategy is that the fillers and solvent are added to the epoxy, and the solvent is then evaporated from the composite by heating it up and thorough mixing. Often, residual amounts of the solvent are found to remain at the interface of the fillers and matrix, rendering almost no improvement in the load and heat transfer.

The method developed here is to covalently functionalize the CNTs with molecules that can take part in the polymerization process of the epoxy resin [24,25] so that strong chemical bonds between the nanotubes and the epoxy matrix can be achieved. The functionalization also causes the CNTs to be slightly polar and thus debundle themselves with the electrostatic interaction. These chemical bonds help to improve the mechanical load transfer between the matrix and the MWNT, and they can also enhance the thermal dissipation due to a better phonon transport through the molecular bridge.

The covalent functionalization is done by grafting molecules that contain an epoxide ring on the MWNTs. Among the various methods that could potentially be developed to bond such molecules, one which does not attack too strongly the CNT lattice, should be used so that the intrinsic properties of the nanotubes are not too affected.

### Method of covalent functionalization

**Figure 1:**
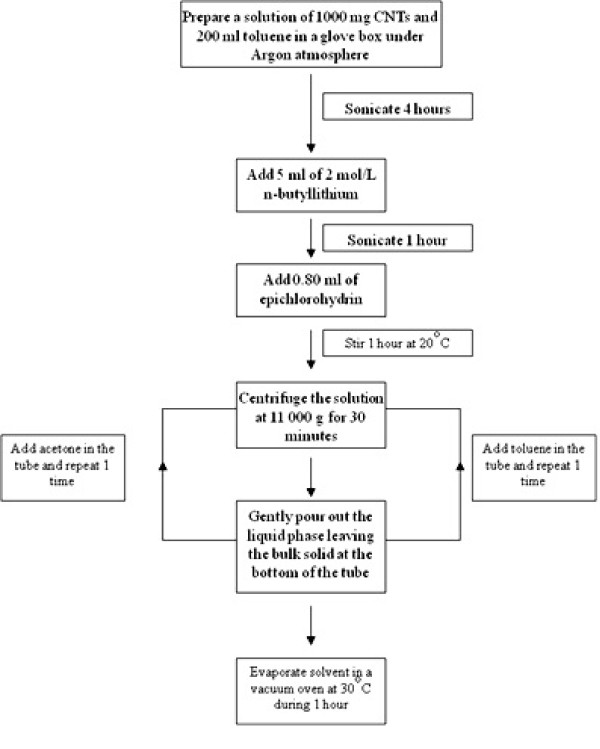
**Flow chart of covalent functionalization method used in this work**.

The method chosen for functionalization in this work is shown in Figure 1. The MWNTs used during the experiments are from Nanothinx Ltd (Nanothinx S.A., Platani, Rio-Patras, Greece). The basic physical characteristics of the CNTs are as follows: purity > 95%, diameter 10 to 20 nm, length 5 to 15 μm, and amorphous carbon 3%.

**Figure 2:**
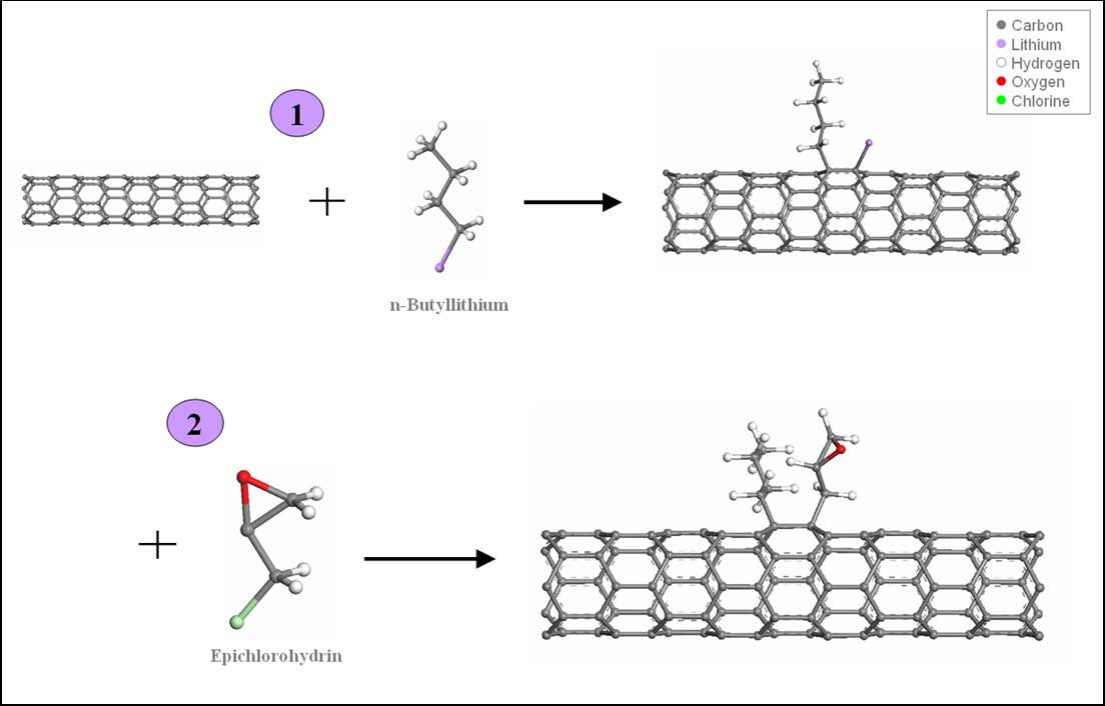
**Chemical reaction for the functionalization**.

As the epoxy to be used in our experiments is EPON828 from Hexion Chemicals (Hexion Specialty Chemicals Inc., Columbus, OH, USA), the molecules that contain the epoxide ring is epichlorohydrin. However, to attach these molecules, broken bonds along the MWNT sidewalls have to be created, and this is done using a highly reactive *n*-butyllithium. The chemical reaction is shown in Figure 2. The covalent nature of the functionalization has been proven in our previous work [25].

During the first phase of the reaction (1 in Figure 2), butyl anions are covalently bonded to the MWNT sidewalls and tips. At some stage in this bonding process, the negative charges are conferred to the butyl-MWNT body. As a result, the butyl-MWNT molecules are negatively charged while the charge balance is ensured by the lithium cations present in the solution. Consequently, the butyl-MWNTs anions naturally repel each other by electrostatic repulsion resulting in CNT debundling. The solution is uniformly black and remains so as long as no other chemical reactions take place. If the container is properly sealed, the CNTs can potentially remain dispersed indefinitely. However, once the second phase of the reaction takes place, the CNTs are electrically neutralized and sediment. Nevertheless, the tendency for bundling is lessened due to the CNT surface non-uniformity resulting from the bonded molecules.

After the functionalization with the conditions as mentioned in Figure 1, the functionalized CNTs [f-CNTs] are filtered out from the reacting solution by centrifugation. During the first filtration run, a yellow solution containing the reacted amorphous carbon, lithium chloride salt, and non-reacted chemicals are separated from the f-CNTs.

To remove any other potential chemical residues, acetone is added to the f-CNT paste, and the mixture is shook thoroughly to ensure a proper homogenization of the f-CNTs and the remaining residual chemicals. Subsequently, the mixture is centrifuged again to separate the f-CNTs from the supernatant.

**Figure 3:**
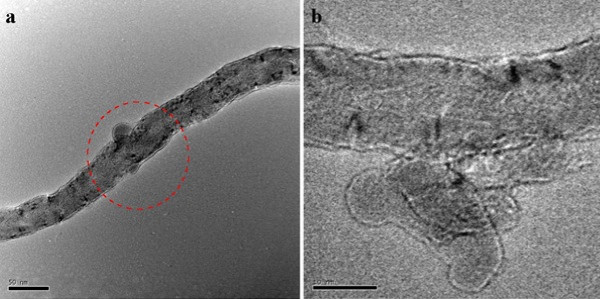
**TEM pictures of f-CNTs**. (**a**) Functionalized site on the CNT sidewall. (**b**) Higher magnification TEM showing molecules bonded to the MWNT sidewall.

Finally, the paste containing only f-CNTs in acetone is placed in a vacuum oven at 30°C to evaporate the acetone. Figure 3 shows the transmission electron microscopy [TEM] photos of these f-CNTs.

### Performance evaluation

**Figure 4:**
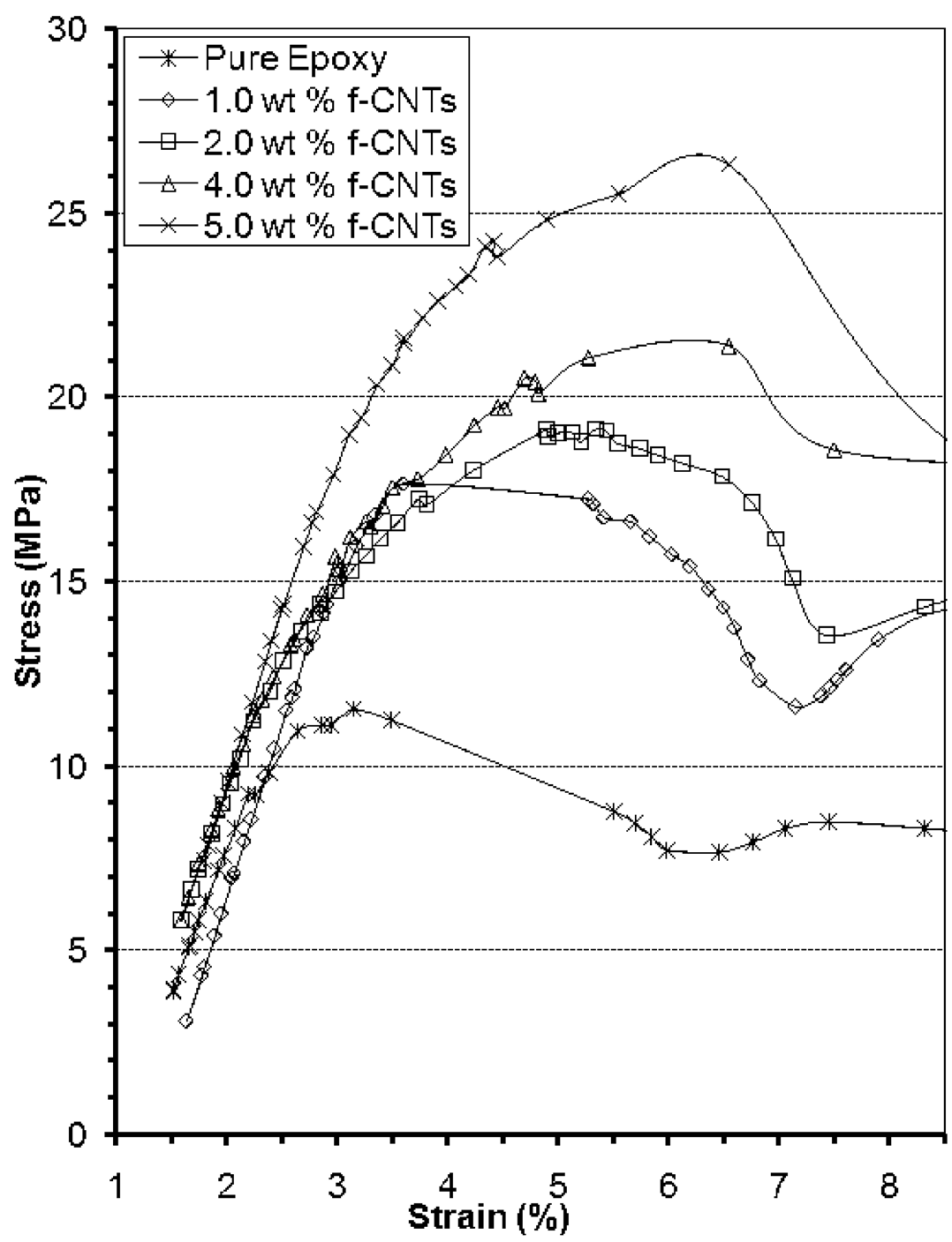
**Tensile characteristics of epoxy composite**.

Experiments are performed by dispersing the above f-CNTs into EPON828 epoxy resin manufactured by Hexion Chemicals. Dispersion is performed mechanically by an overhead stirrer which is a traditional homogenization method extensively used in the manufacturing process of composite materials. During this process, no solvents are used to disperse the CNTs. The f-CNT powder is directly poured in the resin and mixed thoroughly for 4 h. Figure 4 shows the improvement results.

As shown in Figure 4, the room temperature in Young's modulus is about the same for all samples (around 11 ± 1 MPa). However, the tensile strength and the strain at tensile strength vary depending on the amount of CNTs present (Table 1). The physical interpretation for such an occurrence is that the CNTs are homogenously oriented in the composite, and the fraction of CNTs oriented in the direction of the tensile force applied is not sufficient to have an impact on the Young's modulus. However, as the sample is stretched, the CNTs are gradually aligned in the direction under tensile testing and thus reinforce the material against the fracture. This is illustrated schematically in Figure 4. The more CNTs are added to the composite, the bigger is the reinforcement and the higher is the strain.

**Table 1:** Tensile strength and corresponding strain

Percent CNTs	Tensile strength (MPa)	Strain at tensile strength (percent)
0	11.5	2.6

1	17.6	3.0

2	19.1	4.4

4	21.1	4.8

5	25.5	5.0

We also observe a modification of the shape of the curves with an increase in fillers' amount. In fact, the curvature of the lines varies as a function of the amount of CNTs was added above the yield strength. This is explained by the distribution in the orientation, length, diameter, and anchoring of the CNTs, which results in a distribution in the reinforcement capacity of the nanotubes [26].

**Figure 5:**
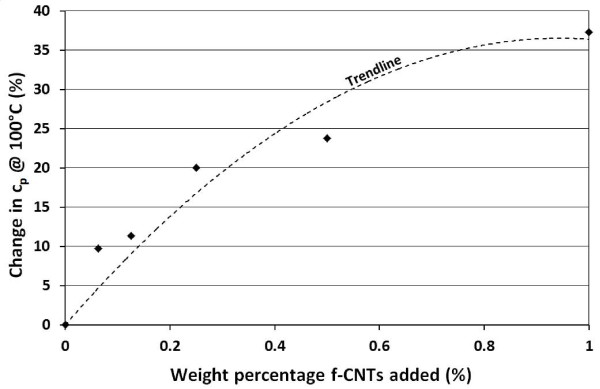
**Evolution of the specific heat capacity, *c*_p _for different amounts of CNTs added**. The black dots are the results measured experimentally and the dotted line, the corresponding trend line.

Figure 5 shows the variation in specific heat capacity of the composite at 100°C under constant pressure [*c*_p_] as a function of the amount of the f-CNTs added. As the thermal conductivity of a material is directly proportional to *c*_p_, the trend line gives an indication of the relative improvement in thermal conductivity of the composite material at different CNT loadings. However, no absolute thermal conductivity measurement is made at this time due to the unavailability of measuring equipment.

### Applications of MWNT to Pb-free solder

Pb-free solder is a necessity for today's soldering material due to the requirement of Reduction of Hazardous Substances directive, and the commonly used Pb-free solder is Sn-Ag-Cu [SAC]. However, such a solder has the following limitations: 1) higher melting points than traditional Sn-Pb solder [27]; 2) poor wettability; and 3) higher coefficient of thermal expansion [CTE] [28]. With the ever-increasing functional requirements and the miniaturization of electronic components, new solder materials which possess a combination of good mechanical, electrical, and thermal properties are desired [29].

Studies have shown that by introducing CNTs into a solder alloy, its overall performance can be improved [30,31]. However, it is also reported that the interaction between Sn and CNT is weak, making a compromised load transfer between the CNTs and the Sn matrix, and hence a limited performance improvement [32].

In order to improve their interaction, Ni coating on CNT is used. Ni is chosen as it can form stable phases (Ni_3_Sn_4_) in the Ni-Sn binary system. Also, Ni has good wetting characteristics with Sn-Ag-Cu solder.

The Ni-coated MWNT [Ni-CNT] is purchased from Tsinghua University, Beijing, China. The method of mixing the Ni-coated MWNT followed the method described by Nai et al. [30]. The method is basically a powder metallurgy route where the powder and CNT are pre-weighted, blended, uniaxially compacted, and finally extruded to form an 8 mm diameter rod.

### Experimental results

**Figure 6:**
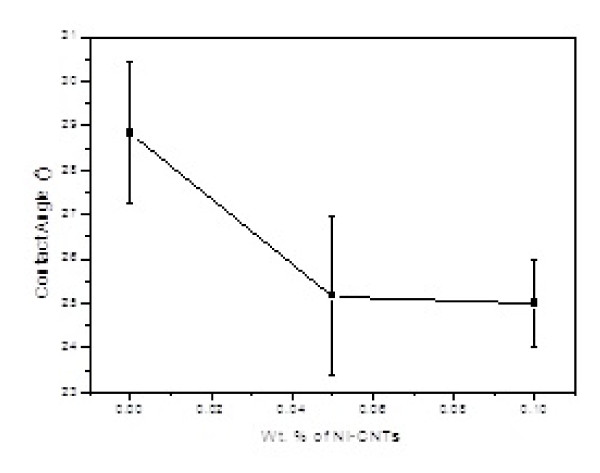
**Improvement in the wettability of solder with Ni-CNT**. The black square is the average contact angle and the lines below and above the black squares are the error bars.

Figure 6 shows the improvement results of wettability due to the embedment of Ni-CNT in the solders [33]. The improvement is believed to be attributed to the lower surface interfacial energy when reinforcements are added [34].

Table 2 shows an improvement in the dimensional stability of the composite solders as compared with their monolithic counterparts. The lowest average CTE value is observed for the case of SAC/0.05Ni-CNT, which exhibits a 5% decrease in value as compared to that of the monolithic SAC samples [29].

**Table 2:** CTE of monolithic and composite solders

Materials	Coefficient of thermal expansion (10^-6^/°C)
SAC	29.15 ± 0.15

SAC/0.05Ni-CNT	27.70 ± 0.10

SAC/0.1Ni-CNT	27.95 ± 0.55

**Figure 7:**
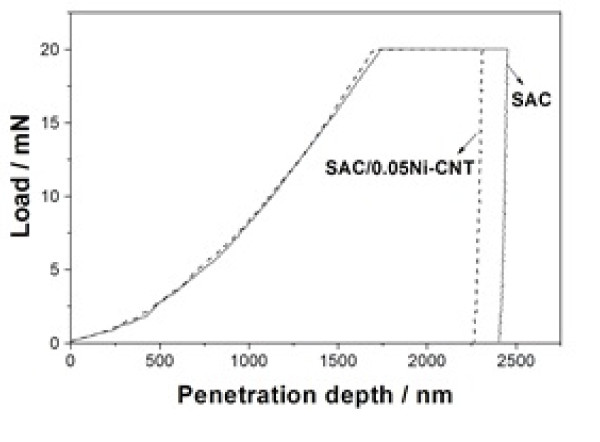
**Typical indentation load-depth curves of SAC and SAC/0.05Ni-CNT solder samples**.

The typical indentation load-depth curves of the SAC and SAC/0.05Ni-CNT solder samples are illustrated in Figure 7. The penetration depths of the SAC solder at the maximum load ranges from 1,740 to 2,450 nm, and that for SAC/0.05Ni-CNT sample ranges from 1,700 to 2,310 nm, indicating a more creep resistant for Ni-CNT than its monolithic counterpart [35].

## Conclusion

In this work, we demonstrated the use of MWNT to improve the thermal and mechanical properties of epoxy using covalent functionalization method for CNT, which establishes a covalent bond between CNTs and the polymer molecules, thus ensuring the flow of phonon for enhanced heat conduction and a strong bond for mechanical strength. We also successfully embed commercially available Ni-coated CNT into Pb-free solder to increases its wettability and mechanical strength. The coefficient of thermal expansion of the modified solder is also reduced through the embedment of Ni-CNT.

These experimental results show the feasibility of using MWNT to improve the packaging material properties to meet the increasing stringent requirements on electronic packaging in order to ensure high reliability and performances of integrated circuits. Further fine tuning will be needed in order for these methods to be widely applied in the electronic packaging industry.

## Competing interests

The method of functionalization presented in this paper is undergoing patent filing. This work is funded by ST Microelectronics for the epoxy portion and by SIMTech for the solder portion.

## Authors' contributions

TCM provided guidance to CB and YH as he was the supervisor. He is also the person who wrote this paper. CB conducted the research study on the chemical functionalization of CNTs for inclusion and homogenization in epoxy resins. He also fabricated and characterized the f-CNT/epoxy composite samples to observe the alterations in mechanical and thermal properties of the epoxy resin due to f-CNT addition. YH conducted the study on the properties of Ni-coated carbon nanotubes reinforced solders. HJ characterized mechanical and thermal properties of the composite solder. All authors read and approved the final manuscript.
